# Factors Associated With Consideration of Future Preference for Medical Aid in Dying in the U.S.

**DOI:** 10.1093/geroni/igaf122.866

**Published:** 2025-12-31

**Authors:** Samuel Nemeth, Elizabeth Luth, Elissa Kozlov

**Affiliations:** Purdue University, West Lafayette, Indiana, United States; Rutgers University, New Brunswick, New Jersey, United States; Rutgers School of Public Health, Piscataway, New Jersey, United States

## Abstract

Medical Aid in Dying (MAID) is a legal medical option for terminally ill patients in 10 U.S. states and Washington DC. Several other states are considering adopting MAID laws. However, it remains unclear what factors predict future preference for using MAID. Using a national convenience sample of 3,221 U.S adults and multinomial logistic regression models, we examine demographic, belief, knowledge, and experience predictors of potential future MAID usage (definitely not [reference], probably not, not sure, probably, definitely). Net of all predictors, Catholicism and Protestantism were both significantly and robustly associated with lowered odds of considering future MAID usage compared to Agnostic/Atheist individuals. For example, Catholicism was associated with 74% lower odds while Protestantism was associated with 79% lower odds of selecting “definitely” (Adjusted Odds Ratio (AOR)=0.26 [95% CI: 0.10, 0.66] and AOR=0.21 [CI: 0.08, 0.53], respectively). Additionally, individuals who believe MAID should not be legal (vs. not sure, AOR=0.48, CI: 0.25, 0.95) were less likely to select “definitely.” Conversely, individuals who were older (AOR=1.01 CI: 1.00, 1.03), believe MAID is moral (vs. not sure, AOR=12.09, CI: 6.23, 23.46) and who had ever euthanized a pet (AOR=2.44 CI: 1.71, 3.48) were more likely to say they definitely would consider pursuing MAID in the future. These findings suggest that preference for considering future MAID usage varies across demographic, belief, and experience factors. While future preferences are not completely indicative of actual behaviors, this study provides insight into possible end-of-life treatment preferences.

